# MicroRNA-92b augments sorafenib resistance in hepatocellular carcinoma via targeting PTEN to activate PI3K/AKT/mTOR signaling

**DOI:** 10.1590/1414-431X2020e10390

**Published:** 2021-05-31

**Authors:** Zhouyang Cheng, Qingfeng Ni, Lei Qin, Yang Shi

**Affiliations:** 1 Department of General Surgery, The First Affiliated Hospital of Soochow University, Soochow, China; 2 Department of General Surgery, Affiliated Hospital of Nantong University, Nantong, China

**Keywords:** MicroRNA-92b, Sorafenib, Resistance, PTEN, Hepatocellular carcinoma

## Abstract

Sorafenib (SOR) resistance is still a significant challenge for the effective treatment of hepatocellular carcinoma (HCC). The mechanism of sorafenib resistance remains unclear. Several microRNAs (miRNAs) have been identified as playing a role in impairing the sensitivity of tumor cells to treatment. We examined the mechanism behind the role of miR-92b in mediating sorafenib resistance in HCC cells. We detected that miR-92b expression was significantly upregulated in SOR-resistant HepG2/SOR cells compared to parental HepG2/WT cells. After transfection with miR-92b inhibitor, the proliferation of HepG2/SOR cells was remarkably weakened and rates of apoptosis significantly increased. PTEN was considered to be a functional target of miR-92b according to a luciferase reporter assay. Knockdown of PTEN significantly impaired the ability of miR-92b inhibitor on increasing sorafenib sensitivity of HepG2/SOR cells. Furthermore, we confirmed by western blotting and immunofluorescence that miR-92b can mediate sorafenib resistance by activating the PI3K/AKT/mTOR pathway in HCC cells by directly targeting PTEN. These findings further validate the mechanism of miR-92b in SOR resistance in HCC treatment.

## Introduction

Hepatocellular carcinoma (HCC) is among the most common malignant tumors. It accounts for approximately 90% of all liver cancers, and remains the major cause of cancer-related death across the world (1,2). The overall survival rate of HCC patients remains low not merely due to the recurrence and metastasis of HCC itself, but also due to a lack of sensitive detection methods and effective treatments (3,4). Currently, the standard therapeutic regimen for advanced HCC patients is molecular targeted therapy (5).

Sorafenib (SOR) is the preferred multi-target kinase inhibitor for treatment of advanced HCC, and can improve overall survival rate of advanced HCC patients (6,7). However, while SOR treatment is initially effective for most patients, it easily loses effectiveness, which indicates that long-term treatment with SOR is related to developing drug resistance (8,9). Acquired SOR resistance involves multiple mechanisms, including mutations of kinase targets, JAK-STAT pathways, crosstalk between PI3K/AKT, epithelial-mesenchymal transition, hypoxia-induced pathways, and others (10,11). Currently, the molecular mechanisms of SOR resistance in HCC remains unknown.

MicroRNAs (miRNAs) are a series of non-coding small RNAs, which, throughout post-transcriptional silencing, play an important role in several biological processes (12). There are also many miRNAs that impair the sensitivity of tumor cells to drugs, and endow them with drug resistance. For example, miR-122 expression leads to the modulation of chemosensitivity of HCC cells, as well as regulation of multidrug resistance-related gene expression (13). Through inhibition of the expression of P-gp in gastric cancer (GC) cells, miR-129 reversed cisplatin-resistance (14). Adriamycin-resistance in breast cancer cells mediated by miR-222 is partly through the PTEN/Akt/FOXO1 signaling pathway (15). miR-340-5p inhibits drug resistance and cell proliferation by increasing apoptosis of breast cancer cells through the Wnt/β-catenin pathway, where expression of LGR5 is down-regulated (16). The proliferation of GC cells is promoted through miR‐92b by up-regulating DAB2IP‐mediated PI3K/AKT signaling pathway (17). In gastric cancer SGC‐7901 cells, miR‐92b is highly expressed. Proliferation, migration, as well as invasion of SGC‐7901 cells, may be inhibited if expression of miR‐92b is interfered with targeting HOXD10 and down-regulation of MMP‐2/9 (18). miR-92b was also reported to be a promotor for HCC progression by targeting Smad7/lncRNA XIST axis (19). Nevertheless, it is still unclear whether miR-92b also participates in the resistance to SOR in HCC cells.

In this study, we set up a SOR-resistant cell model and conducted a significant investigation into the correlation between SOR resistance and miR-92b expression, demonstrating that miR-92b/PTEN axis can regulate SOR sensitivity of HCC cells via the PI3K/AKT/mTOR signaling pathway.

## Material and Methods

### Cell culture and development of SOR-resistant cells

We purchased the human HepG2 cell line from American Type Culture Collection (USA). We established the HepG2/SOR cell line using the parental HepG2 cells from our laboratory by exposing our cell line to increasing concentrations of SOR (MedChem Express, USA), ranging from 0.5-10 µM for six months. Specifically, the concentration of SOR increased by 0.5 µM every week, the well-conditioned cells were passed to the following induction. When the concentration of SOR ranged between 8 to 10 µM, the internal induction was prolonged to 2 weeks. Finally, the cells that survived well in the 10-µM SOR medium were collected and maintained in DMEM supplemented with 10% FBS and 10 µM SOR in a 37°C humidified atmosphere with 5% CO_2_ in order to develop the HepG2/SOR cell line. Prior to conducting the following experiments, HepG2/SOR cells were cultured in medium without SOR for one week.

### Cell viability by MTT assay

We used 3-(4,5)-dimethylthiazol (-z-y1)-3,5-di-phenyltetrazoliumbromide MTT assay to determine relative cell viability. After 48 h of SOR treatment in the absence or presence of transfection, we incubated cells at a density of 5000 cells/well with 5 μg/mL MTT at 37°C for 4 h. We measured absorbance values at 570 nm with a spectrophotometer (Thermo, USA). All assays were performed in triplicates.

### Cell transfection

The human miR-92b inhibitor, miR-92b mimics, respective negative controls (NC), si-PTEN, as well as negative control (si-NC), were designed and purchased from GenePharma (China), and were co-cultured with Lipofectamine 2000 in accordance with the manufacturer's protocol when cells reached 30-50% confluence.

### RNA isolation and qRT‐PCR

The expression of miR‐92b and PTEN were measured using quantitative real-time PCR (qRT‐PCR). Total RNA isolation was conducted according to previous reports (20). The miR‐92b expression was normalized to U6, and PTEN was normalized to GAPDH. The assays were performed in triplicates. The sequences of the primers were: miR-92b forward: 5′-TATTGCACTCGTCCCGGCCTCC-3′ and reverse: 5′-GTGCAGGGTCCGAGGT-3′; PTEN forward: 5′-CCCAGTCAGAGGCGCTATGTGTAT-3′ and reverse: 5′-GTTCCGCCACTGAACATTGG-3′; U6 forward: 5′-GTGCTCGCTTCGGCAGCACATATAC-3′ and reverse: 5′-AAAAATATGGAACGCTCACGAATTTG-3′; GAPDH forward: 5′-ATGTCGTGGAGTCTACTGGC-3′ and reverse: 5′-TGACCTTGCCCACAGCCTTG-3′.

### Flow cytometric analysis of cell apoptosis

Annexin V-FITC apoptosis detection kit (Beyotime, China) was used for evaluation of cellular apoptosis. Following the treatment, cells were trypsinized and washed with ice-cold PBS. Cell suspensions were double stained with Annexin V-FITC and PI at room temperature in the dark for 15 min. The cells underwent analysis by flow cytometry (BD Bioscience, USA). The assays were performed in triplicate.

### Protein extraction and western blotting

Protein extraction and western blotting were conducted as reported before (20). Antibodies against PTEN, GAPDH, p‐AKT, p-mTOR, AKT, and mTOR were provided by Abcam (UK). GAPDH was used as internal control. The assays were performed in triplicate.

### Luciferase reporter assay

We synthesized the 3′-UTR sequence of PTEN, which was predicted to target miR-92b, and inserted it into a pmiR-GLO dual-luciferase miRNA target expression vector, together with a corresponding mutated sequence. Subsequently, we co-transfected HepG2/SOR cells with mutant or wild-type 3′-UTR of PTEN vector using a Lipofectamine 2000 reagent. We harvested the cells after 48 h and measured them in accordance with the manufacturer's instructions. The ratio between the Renilla and firefly luciferase activities was defined as relative luciferase activity. The assays were performed in triplicate.

### Immunofluorescence assay

After reaching over 80% confluence, HepG2/SOR cells were washed with PBS, and cold methanol was added for 20-min to fix cells. The cells were then permeabilized with 0.1% Triton X-100 for 10 min and then washed with PBS. Cells were then treated with primary antibody against p-AKT or p-mTOR (Abcam) overnight at 4°C in PBS with 1% BSA. Next, secondary Alexa Fluor® 594-conjugated antibody (Cell Signaling Technology, USA) was added for 1 h at room temperature. The nuclei were stained with DAPI (Thermo Fisher Scientific, USA) for 5 min, and then the coverslips were mounted on glass slides for immunofluorescence analysis with a Leica fluorescence microscope (Germany).

### Statistical methods

Data was analyzed with GraphPad Prism 7.0 (USA) and SPSS software 16.0 (IBM, USA). Data are reported as means±SD. A one-way ANOVA was utilized to compare the multiple groups. Student's *t*-test was utilized to compare between the two groups. P<0.05 indicates a significant statistical difference.

## Results

### miR-92b was overexpressed in SOR-resistant HepG2 cells

**Figure 1:**
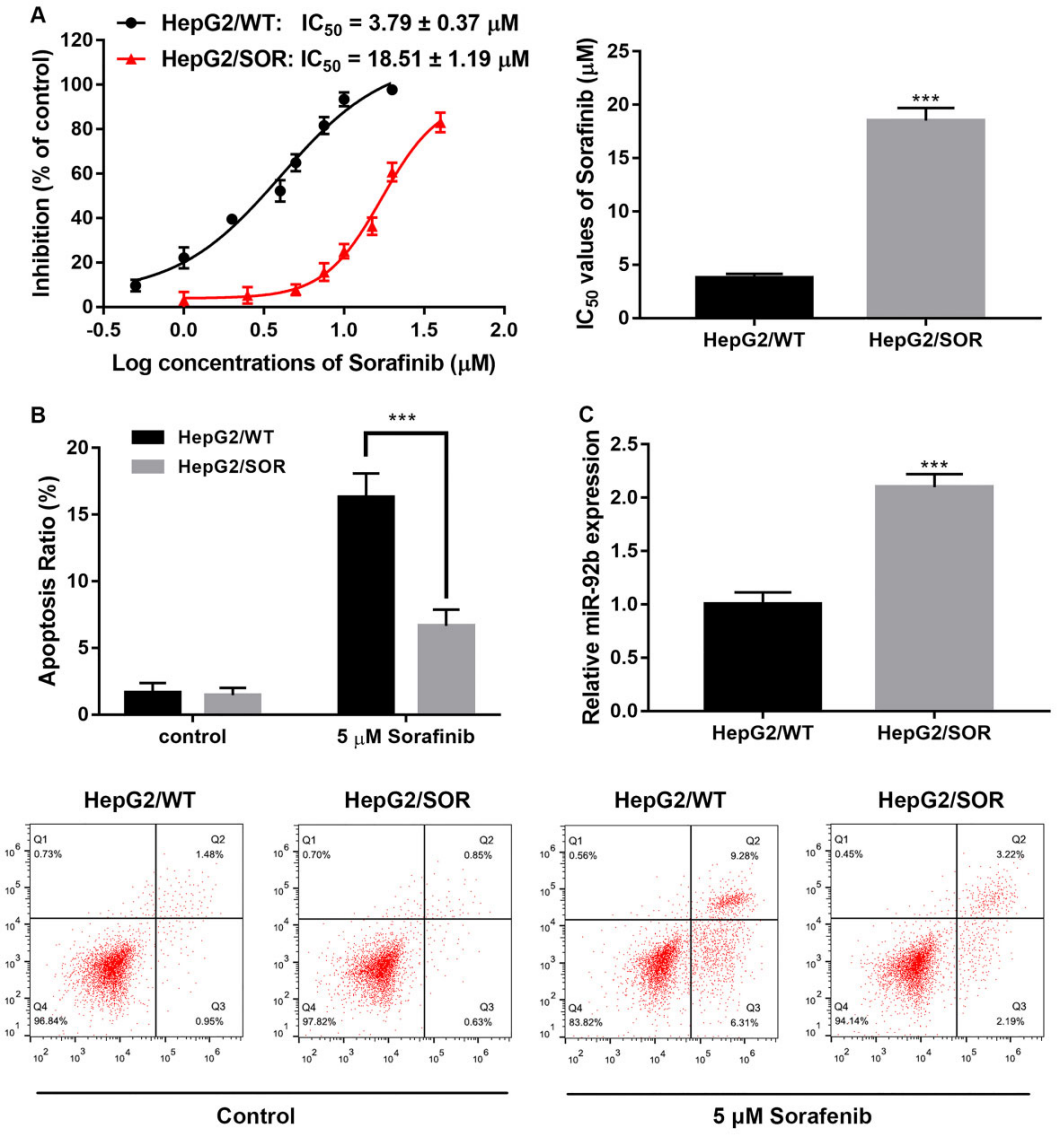
miR-92b is overexpressed in sorafenib-resistant HepG2 cells. **A**, Rates of inhibition and half maximal inhibitory concentration (IC_50_) values of sorafenib (SOR) in HepG2/SOR or HepG2/WT (wild type) cells. **B**, Flow cytometry was conducted to measure the apoptosis of HepG2/SOR and HepG2/WT cells treated with SOR (0.5 μM). **C**, qRT-PCR analysis of miR-92b expression in HepG2/WT or HepG2/SOR cells. Data are reported as means±SD, ***P<0.001 (Student's *t*-test).

In order to study the mechanism of SOR-resistance, we developed HepG2/SOR cells. In order to test for SOR resistance ability, the MTT assay helped determine half maximal inhibitory concentration (IC_50_) values of HepG2/SOR cells and SOR-treated HepG2/WT cells. The IC_50_ value of SOR in HepG2/SOR cells was 18.51±1.19 μM, which was significantly higher than that in HepG2/WT cells (3.79±0.37 μM) (Figure 1A, P<0.001). Additionally, flow cytometry demonstrated that there was significantly less apoptosis in HepG2/SOR cells treated with 5 μM SOR compared to HepG2/WT cells, suggesting the successful establishment of SOR resistance (Figure 1B). Then, we investigated miR-92b expression in HepG2/WT and HepG2/SOR cells. Results demonstrated that miR-92b was significantly overexpressed in HepG2/SOR cells versus the parental counterparts (Figure 1C).

### Down-regulation of miR-92b increased the SOR sensitivity of HepG2/SOR cells

**Figure 2:**
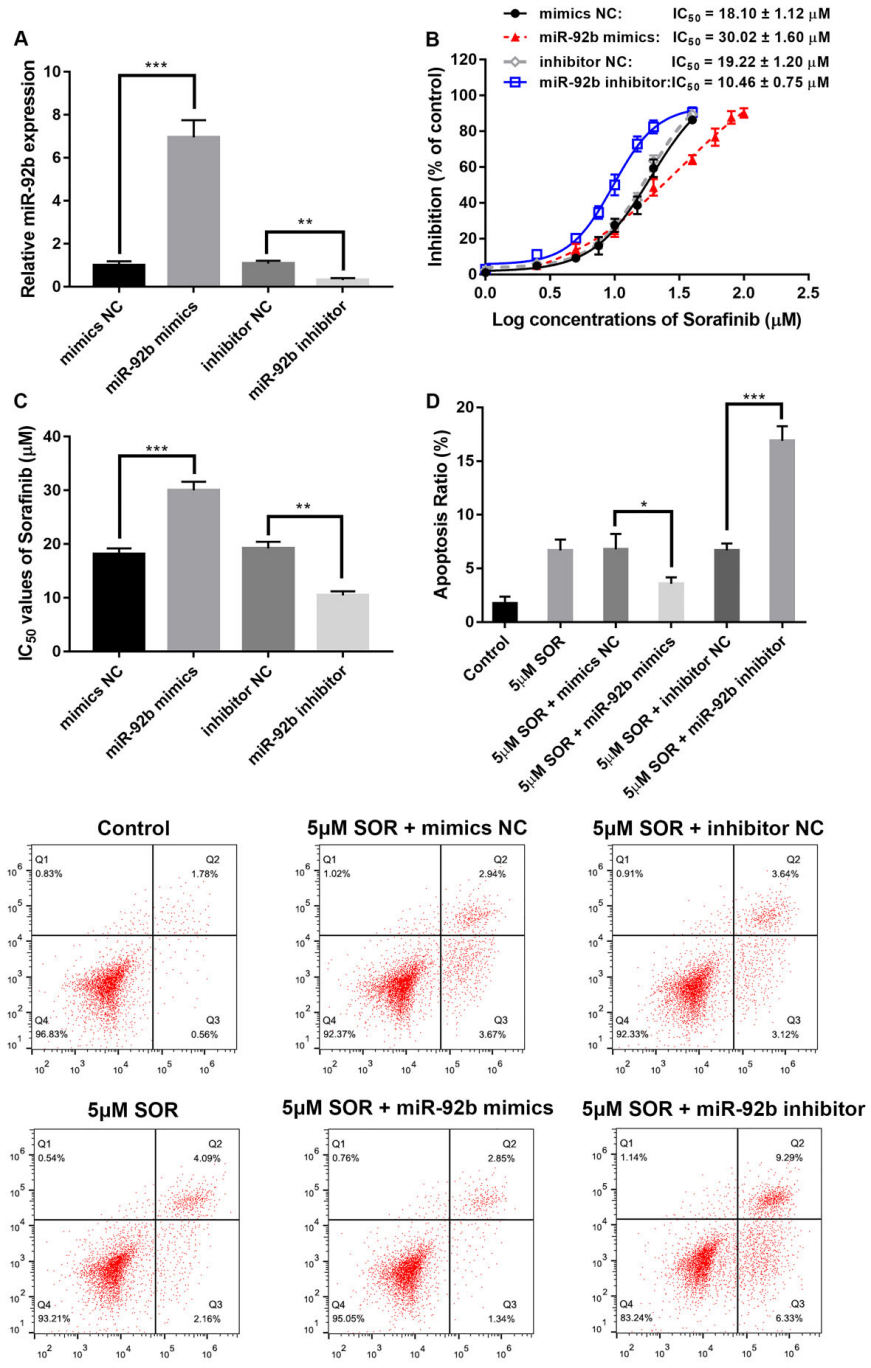
Down-regulation of miR-92b increases sorafenib (SOR) sensitivity in HepG2/SOR cells. **A**, qRT-PCR analysis of miR-92b expression at different transfections. **B** and **C**, Rates of inhibition and half maximal inhibitory concentration (IC_50_) values of SOR in HepG2/SOR cells. **D**, Flow cytometry measured apoptosis rates of HepG2/SOR cells at different transfections and incubation with SOR (0.5 μM). Data are reported as means±SD. *P<0.05, **P<0.01, ***P<0.001 (ANOVA). NC: negative control.

qRT-PCR confirmed high expression of miR-92b after miR-92b mimics transfection, while transfection of miR-92b inhibitor led to the opposite effect (Figure 2A). MTT assay results indicated significantly reduced IC_50_ values of SOR after miR-92b inhibitor transfection, while miR-92b mimics transfection led to a significant increase in IC_50_ values (Figure 2B and C). Down-regulation of miR-92b also significantly promoted SOR-induced cellular apoptosis, while up-regulation of miR-92b significantly inhibited apoptosis (Figure 2D).

### miR-92b targeted 3′UTR of PTEN

**Figure 3:**
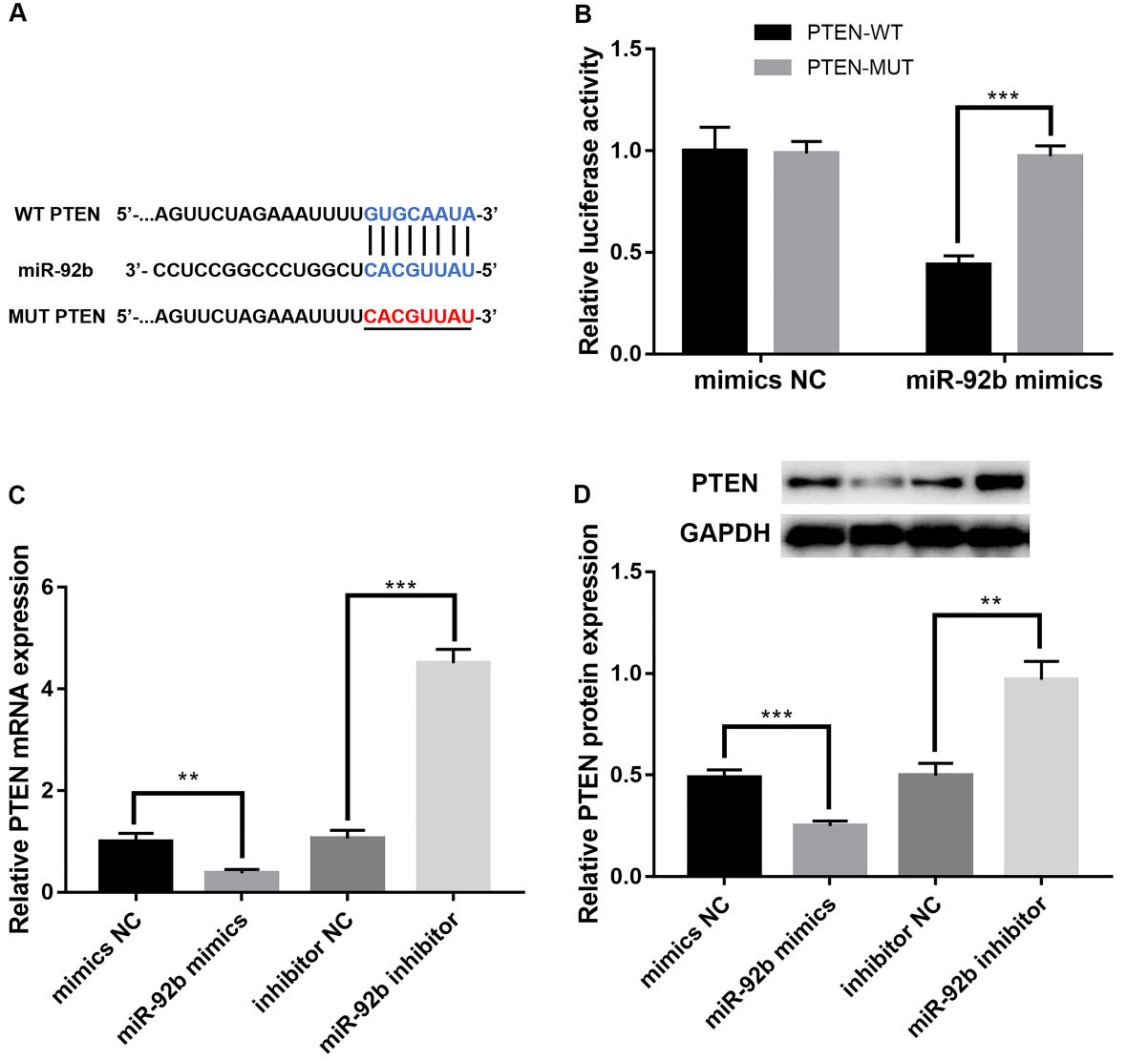
PTEN is a target of miR-92b. **A**, Binding site of miR-92b is located on PTEN mRNA 3′UTR. **B**, Relative luciferase activities in HepG2/SOR cells co-transfected with miR-92b mimics and luciferase reporters containing PTEN-WT (wild type) or PTEN-MUT (mutant) transcript. **C**, The mRNA expression and (**D**) PTEN protein expression was measured using qRT-PCR and western blot in HepG2/SOR cells at different transfections. Data are reported as means±SD. **P<0.01, ***P<0.001 (ANOVA and Student's *t*-test). NC: negative control.

In order to ascertain the potential mechanisms of miR-92b in regulating SOR resistance in HCC, TargetScan (http://www.targetscan.org) and miRBase (http://www.mirbase.org) databases were utilized to predict possible binding sites of miR-92b. Potential target for miR-92b was found to be in the 3′UTR region of PTEN mRNA (Figure 3A). The results of the luciferase assay demonstrated that miR-92b lowers luciferase activity of PTEN-WT but not of PTEN-MUT in HepG2/SOR cells (Figure 3B). Furthermore, western blot and qRT-PCR revealed that over-expression of miR-92b in HepG2/SOR cells led to decreased expression of PTEN, while down-regulation of miR-92b led to the opposite effect (Figure 3C and D).

### Effects of miR-92b on HepG2/SOR cells were partially reversed by PTEN

**Figure 4:**
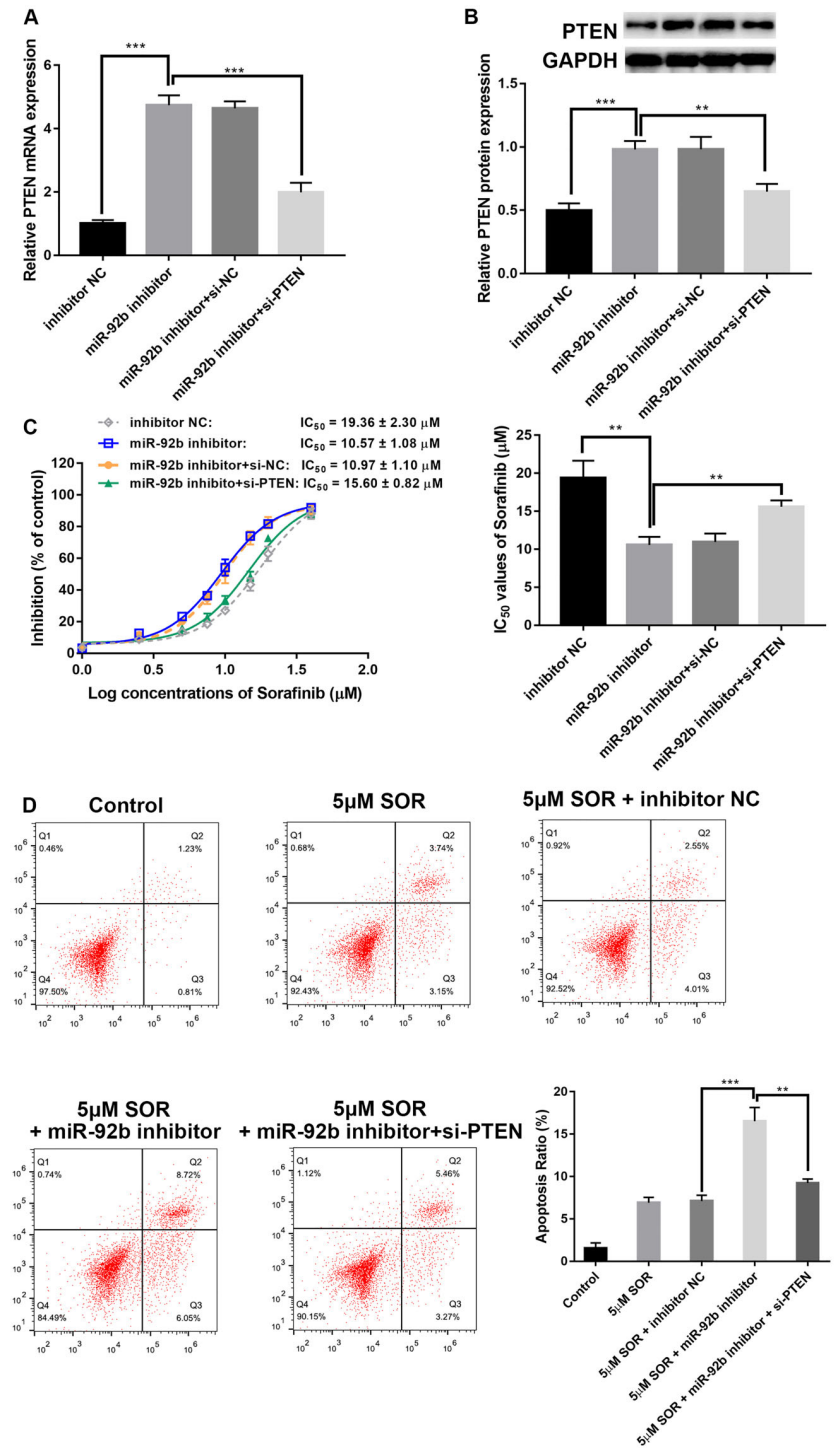
Knockdown of PTEN partially reversed the effect of miR-92b inhibitor in HepG2/SOR cells. **A**, mRNA expression and (**B**) PTEN protein expression were measured using qRT-PCR and western blot in HepG2/SOR cells at different transfections. **C**, Rates of inhibition and half maximal inhibitory concentration (IC_50_) values of sorafenib in HepG2/SOR cells at different transfections. **D**, Flow cytometry was performed to confirm the effect of PTEN on apoptosis of miR-92b-downregulated HepG2/SOR cells under sorafenib treatment. Data are reported as means±SD. **P<0.01, ***P<0.001 (ANOVA). NC: negative control.

In order to further confirm that miR-92b exerts biological potency by targeting PTEN, we co-transfected HepG2/SOR cells with miR-92b inhibitor and PTEN siRNA (si-PTEN). The results demonstrated that knockdown of PTEN significantly decreased mRNA and protein expression of PTEN (Figure 4A and B). Knockdown of PTEN partially abrogated the impact of miR-92b inhibitor, resulting in increased IC_50_ values of SOR (Figure 4C) and significantly reduced apoptosis with SOR (Figure 4D).

### Down-regulation of miR-92b suppressed PI3K/AKT/mTOR signaling pathways in HepG2/SOR cells

**Figure 5:**
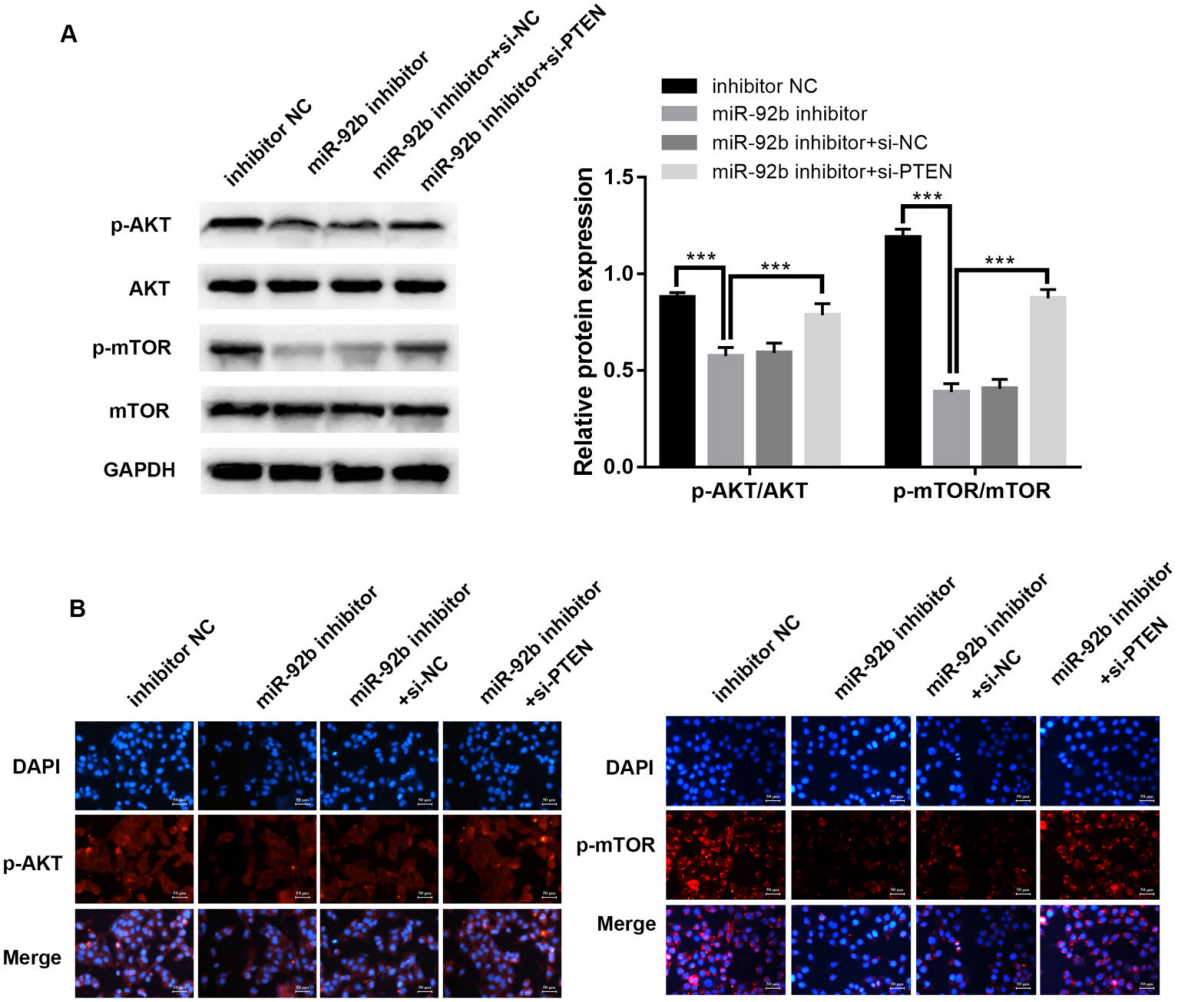
Knockdown of miR-92b suppresses PI3K/AKT/mTOR signaling pathways in HepG2/SOR cells and down-regulation of PTEN reverses the effect. **A**, Western blot analysis of p-mTOR, mTOR, p-AKT, and AKT. **B**, Representative immunofluorescence photomicrograph of p-AKT and p-mTOR (red). Nuclei were stained with DAPI (blue). Scale bar: 50 μm. Data are reported as means±SD. ***P<0.001 (ANOVA). NC: negative control.

In order to identify the molecular mechanism of miR-92b, we investigated expression of key proteins in the PI3K/AKT/mTOR signaling pathways. Western blot (Figure 5A, P<0.001) and immunofluorescence (Figure 5B) assays demonstrated that phosphorylation of mTOR and AKT were dramatically attenuated in HepG2/SOR cells after transfection with miR-92b inhibitor compared to the negative control group. Furthermore, protein expression of p-mTOR and p-AKT were significantly increased by PTEN knockdown, reversing the impact of miR-92b inhibitor.

### Inhibition of p-mTOR attenuated SOR resistance of HepG2/SOR cells induced by miR‐92b

**Figure 6:**
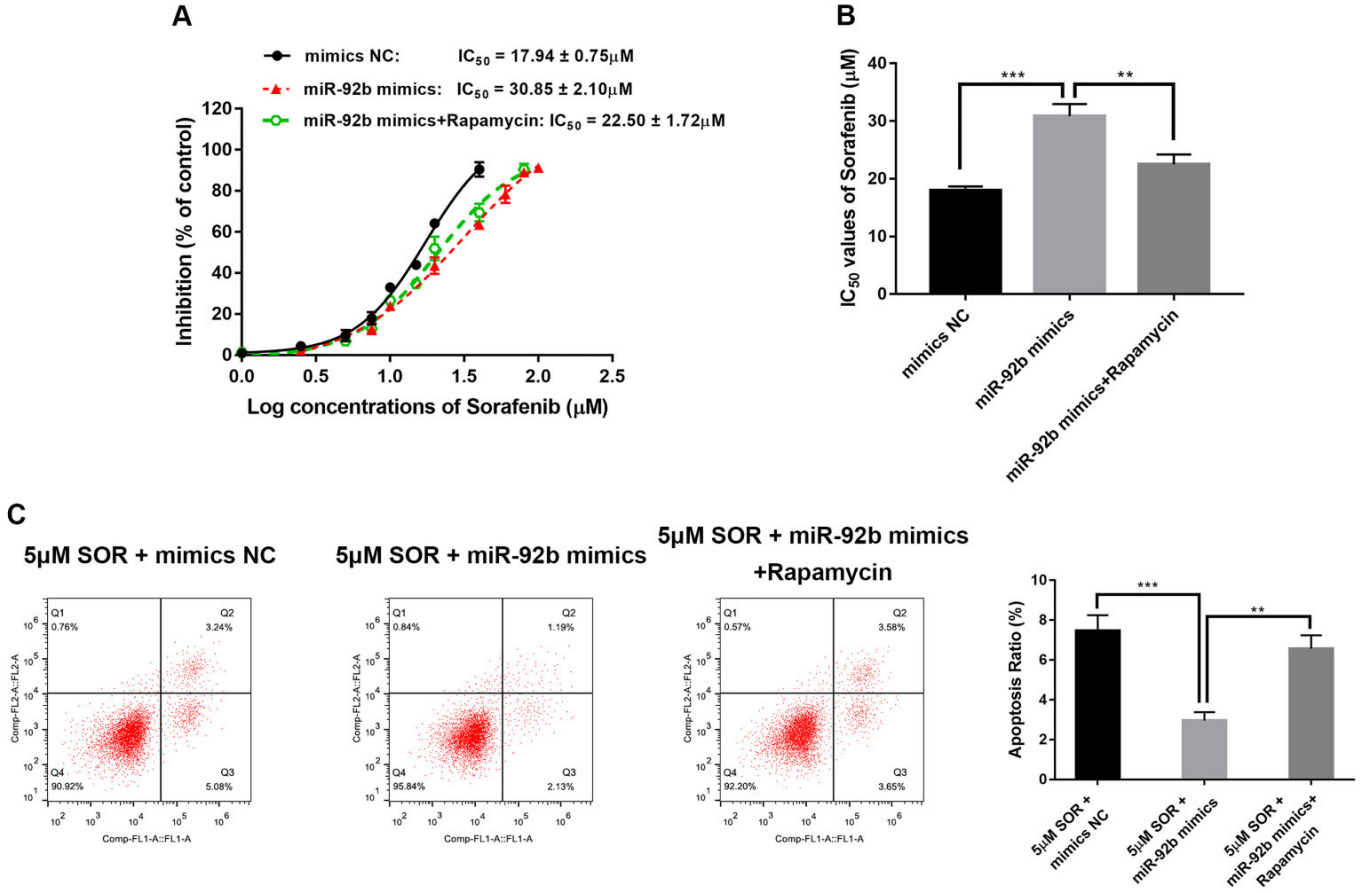
Inhibition of p-mTOR attenuated the sorafenib resistance of HepG2/SOR cells induced by miR-92b. **A**, Rates of inhibition and (**B**) half maximal inhibitory concentration (IC_50_) values of sorafenib in HepG2/SOR cells transfected with mimics negative control (NC), miR-92b mimics, or miR-92b mimics+rapamycin. **C**, Flow cytometry was conducted to measure apoptosis of HepG2/SOR cells transfected with mimics NC, miR-92b mimics, or miR-92b mimics+rapamycin. Data are reported as means±SD. **P<0.01, ***P<0.001 (ANOVA).

We used the specific mTOR inhibitor, rapamycin (MedChemExpress, USA), to explore whether the PI3K/AKT/mTOR signaling pathway participates in SOR resistance induced by miR‐92b. Rapamycin reversed the impact of miR-92b mimics, resulting in a significant decrease in IC_50_ values of SOR (Figure 6A and B) and dramatically increased apoptosis with SOR (Figure 6C).

## Discussion

Further exploration of SOR helps advance the fight against advanced HCC by molecular targeted therapy. Unfortunately, this potential new treatment method leads to low survival benefits. Several HCC patients initially responded to SOR, but eventually the disease progresses and treatment fails (21). Relatively fast acquired resistance to anticancer drugs remains a major problem to cancer treatment (22). The molecular mechanism that lies in SOR resistance is very complex, and remains poorly understood. Therefore, it is necessary to study the potential mechanisms of acquired SOR resistance, as well as to explore promising strategies to improve its efficacy on HCC.

Several studies about molecular mechanisms have revealed that miRNAs may regulate various signaling pathways (23,24). Among numerous *in vivo*, *in vitro*, and patient studies, several miRNAs have been reportedly dysregulated. That is, the fact that they are downregulated or upregulated suggests that it may have an effect on SOR resistance (25). Here, we studied the relationship between SOR resistance and miR-92b expression in HCC cells.

miR-92b was previously regarded as a promising prognostic and diagnostic biomarker for several malignancies, including gastric cancer (26), pancreatic cancer (27), bladder cancer (28), glioblastomas (29), and esophageal squamous cell carcinoma (30). Additionally, it has been reported by Li et al. (31) that miR-92b targets PTEN, and is involved in chemosensitivity of A549 NSCLC cell line to cisplatin. Our study revealed that miR-92b expression was related to the SOR sensitivity of HepG2 cells.

In this study, SOR-resistant HepG2 cells were developed, which was demonstrated by the MTT and apoptosis assays. miR-92b expression was significantly upregulated in HepG2/SOR cells compared to parental HepG2/WT cells. After miR-92b inhibitor transfection, the proliferation of HepG2/SOR cells was remarkably weakened and apoptosis-promoting ability was significantly strengthened, whereas miR-92b mimics had the opposite effect in HepG2/SOR cells. To further assess the function of miR-92b, the target gene of miR-92b was explored, and PTEN was considered to be a functional target of miR-92b.

PTEN is known to be a tumor suppressor with multiple functions, showing frequent loss in many human cancers, including HCC (32). Using luciferase experiments, we found that PTEN is a promising target for miR-92b. miR-92b mimics significantly downregulated PTEN, and miR-92b inhibitor had the opposite effect. Moreover, PTEN siRNA significantly impaired the impact of miR-92b inhibitor on boosting SOR sensitivity of HepG2/SOR cells. These findings strongly suggest that by targeting PTEN, miR-92b regulated resistance to SOR in HCC cells.

The PI3K/AKT pathway plays a key role in SOR resistance as it crosses with the main SOR-targeting MAPK/ERK pathway (33). The PI3K pathway can regulate survival signal to prevent apoptosis and promote carcinogenesis of HCC cells (34). PTEN participates in cell cycle, cell proliferation, adhesion, migration, invasion, metastasis, and apoptosis during cancer development and progression (35). Therefore, downregulation of PTEN leads to activation of PI3K/AKT signaling pathway and promotes cellular proliferation and anti-apoptosis among various cancer cells (35,36).

It has been confirmed by western blotting and immunofluorescence that low expression of miR-92b hinders phosphorylation of AKT and mTOR, while knockdown of PTEN expression at the same time weakened this inhibitory effect. Furthermore, the mTOR inhibitor rapamycin reversed the impact of miR-92b mimics. Taken together, these data suggested that miR-92b could mediate SOR resistance by activation of PI3K/AKT/mTOR pathway in HCC cells by directly targeting PTEN. Our study indicated that miR-92b may be a promising candidate target for reversing SOR resistance in HCC treatment.

In summary, we found that miR-92b was highly expressed in SOR-resistant HCC cells, and contributed to the SOR resistance of HCC cells by targeting PTEN and regulating the PI3K/AKT/mTOR pathway.
